# Coregulator profiling of the glucocorticoid receptor in lymphoid malignancies

**DOI:** 10.18632/oncotarget.22764

**Published:** 2017-11-30

**Authors:** Dorien Clarisse, Jonathan Thommis, Karlien Van Wesemael, René Houtman, Dariusz Ratman, Jan Tavernier, Fritz Offner, Ilse Beck, Karolien De Bosscher

**Affiliations:** ^1^ Receptor Research Laboratories, Nuclear Receptor Lab (NRL) and Cytokine Receptor Lab (CRL), VIB-UGent Center for Medical Biotechnology, Ghent University, Ghent, Belgium; ^2^ Laboratory of Experimental Cancer Research (LECR), Department of Radiation Oncology and Experimental Cancer Research, Ghent University, Ghent, Belgium; ^3^ Cancer Research Institute Ghent (CRIG), Ghent, Belgium; ^4^ Hematology, Department of Internal Medicine, Ghent University Hospital, Ghent, Belgium; ^5^ PamGene International B.V., ‘s Hertogenbosch, The Netherlands; ^6^ Current/Present address: Roche Global IT Solutions, Roche-Polska, Warsaw, Poland; ^7^ Department of Health Sciences, Odisee University College, Ghent, Belgium

**Keywords:** coregulator, glucocorticoid, glucocorticoid receptor, multiple myeloma, acute lymphoblastic leukemia

## Abstract

Coregulators cooperate with nuclear receptors, such as the glucocorticoid receptor (GR), to enhance or repress transcription. These regulatory proteins are implicated in cancer, yet, their role in lymphoid malignancies, including multiple myeloma (MM) and acute lymphoblastic leukemia (ALL), is largely unknown. Here, we report the use and extension of the microarray assay for real-time nuclear receptor coregulator interactions (MARCoNI) technology to detect coregulator associations with endogenous GR in cell lysates. We use MARCoNI to determine the GR coregulator profile of glucocorticoid-sensitive (MM and ALL) and glucocorticoid-resistant (ALL) cells, and identify common and unique coregulators for different cell line comparisons. Overall, we identify SRC-1/2/3, PGC-1α, RIP140 and DAX-1 as the strongest interacting coregulators of GR in MM and ALL cells and show that the interaction strength does not correlate with GR protein levels. Lastly, as a step towards patient samples, we determine the GR coregulator profile of peripheral blood mononuclear cells. We profile the interactions between GR and coregulators in MM and ALL cells and suggest to further explore the GR coregulator profile in hematological patient samples.

## INTRODUCTION

Coregulators are proteins that interact with nuclear receptors (NRs) and other transcription factors (TFs) to modulate gene transcription [1]. They are generally divided into two groups: coactivators, that interact (mostly) with agonist-bound NRs to promote gene transcription, and corepressors, that bind unliganded or antagonist-bound NRs to inhibit gene transcription [2, 3]. However, this strict separation needs to be nuanced, since coactivators can act as corepressors and vice versa depending on the post-translational modification (PTM) status of the coregulator, the NR and the promoter context [4, 5]. Coregulators are intricately involved in different physiological processes such as growth and development, reproduction and energy homeostasis, but also in pathological processes, including metabolic and reproductive diseases and cancers [4, 6].

Since transcription initiation is an ordered and dynamic process, coregulators with different structure and functions are essential [2] and so far, more than 400 coregulators have been identified [7]. The first discovered NR coactivators were the steroid receptor coactivator (SRC) family, consisting of three members: SRC-1 (NCOA1), SRC-2 (NCOA2/GRIP1/TIF2) and SRC-3 (NCOA3/AIB1) [8]. Upon NR activation, SRCs are recruited to target gene promoters and act as adaptors, resulting in the formation of coactivator-dependent multiprotein complexes [2, 3]. To this end, SRCs, harboring weak intrinsic histone acetyltransferase activity, interact with other histone tail modifying coactivators such as histone acetyltransferases (HAT), e.g. cyclic AMP response-element binding protein (CREB)-binding protein (CBP) and p300 (a 300-kD homologue to CBP), and histone methyltransferases (HMT), e.g. co-activator associated methyltransferase 1 (CARM1) [9]. SRCs also engage the transcriptional mediator complex MED1 and recruit co-coactivators such as chromatin remodelers. In addition, SRC PTMs, including phosphorylation, acetylation and methylation, contribute to the association of the coactivator complex and affect the recruitment of the general TFs and RNA polymerase II [4, 9, 10].

Coactivators can also act as signal integrators [1]. In response to extracellular signals such as growth factors (e.g. epidermal growth factor (EGF)), cytokines (e.g. IL-6) and steroid hormones, downstream signaling pathways are activated that utilize kinase cascades to phosphorylate coactivators such as SRCs. Depending on the SRC phosphorylation pattern, specific NRs or TFs and other coregulators are attracted to stimulate gene expression [9].

The glucocorticoid receptor (GR) is part of the NR superfamily and is stimulated by glucocorticoids (GCs). Upon activation, GR translocates to the nucleus where it recruits coregulators [11]. The mechanism by which GR modulates gene expression remains a heavily debated and controversial theme [12, 13]. As two extremes of many different mechanisms, activated GR can enhance the transcription of target genes (transactivation), or repress TF-driven (NF-κB, AP-1) gene expression (transrepression) [14, 15] at specific gene promoters. However, also non-genomic mechanisms are described, including the modulation of signaling pathways, e.g. MAPK by membrane-bound GR [16, 17].

GCs play a role in inflammation, immunity, metabolism, development, reproduction and cognition [18]. Therapeutically they are used in inflammatory and auto-immune disorders, but also in cancer [18]. In lymphoid malignancies such as multiple myeloma (MM) and acute lymphoblastic leukemia (ALL), GCs are an integral part of the treatment strategy [19]. In contrast to MM, which is a hematological disorder of terminally differentiated plasma cells and is localized in the bone marrow, ALL resides in the blood and is characterized by an uncontrolled proliferation of immature lymphocytes [20, 21]. Although GCs efficiently induce apoptosis of these malignant MM and ALL cells, prolonged administration of GCs entails two major limitations. First, there are the side-effects including e.g. diabetes, osteoporosis and edema [22], and second there is the emergence of GC resistance, of which the underlying mechanisms are manifold and often cell-type specific [19, 23, 24]. For instance, GR levels are important determinants of GC sensitivity and resistance and nuclear receptor corepressor 1 (NCOR1) was shown to contribute to the GC-induced GR gene repression mechanism and by extension to GC resistance [25].

In this study, we extended and used the microarray assay for real-time coregulator nuclear receptor interactions (MARCoNI) technology to monitor coregulator associations with endogenous GR of GC-sensitive MM and ALL cells, GC-resistant ALL cells and lung carcinoma cells. MARCoNI uses PamChip arrays onto which 154 coregulator-derived peptides were spotted, each containing an NR-binding motif, including LXXLL for coactivators or LXXXIXXXL for corepressors, allowing the simultaneous detection of 67 coregulator interactions [26]. This technology was originally developed for recombinant or overexpressed NRs, but we extended its use to identify the coregulator profile of endogenous GR from a cellular context, which was, up till now, only reported for estrogen receptor ɑ (ERɑ) [27]. We identified common and unique coregulators in different cell line comparisons using MARCoNI and validated the results using co-immunoprecipitation. Signal intensities, quantifying the interaction between GR and a certain coregulator, were linked back to GR protein levels. As a step towards patient samples, we also determined the GR coregulator profile of peripheral blood mononuclear cells (PBMCs).

## RESULTS

### Coregulator profiling of MM and ALL cell lines using MARCoNI

**Figure 1:**
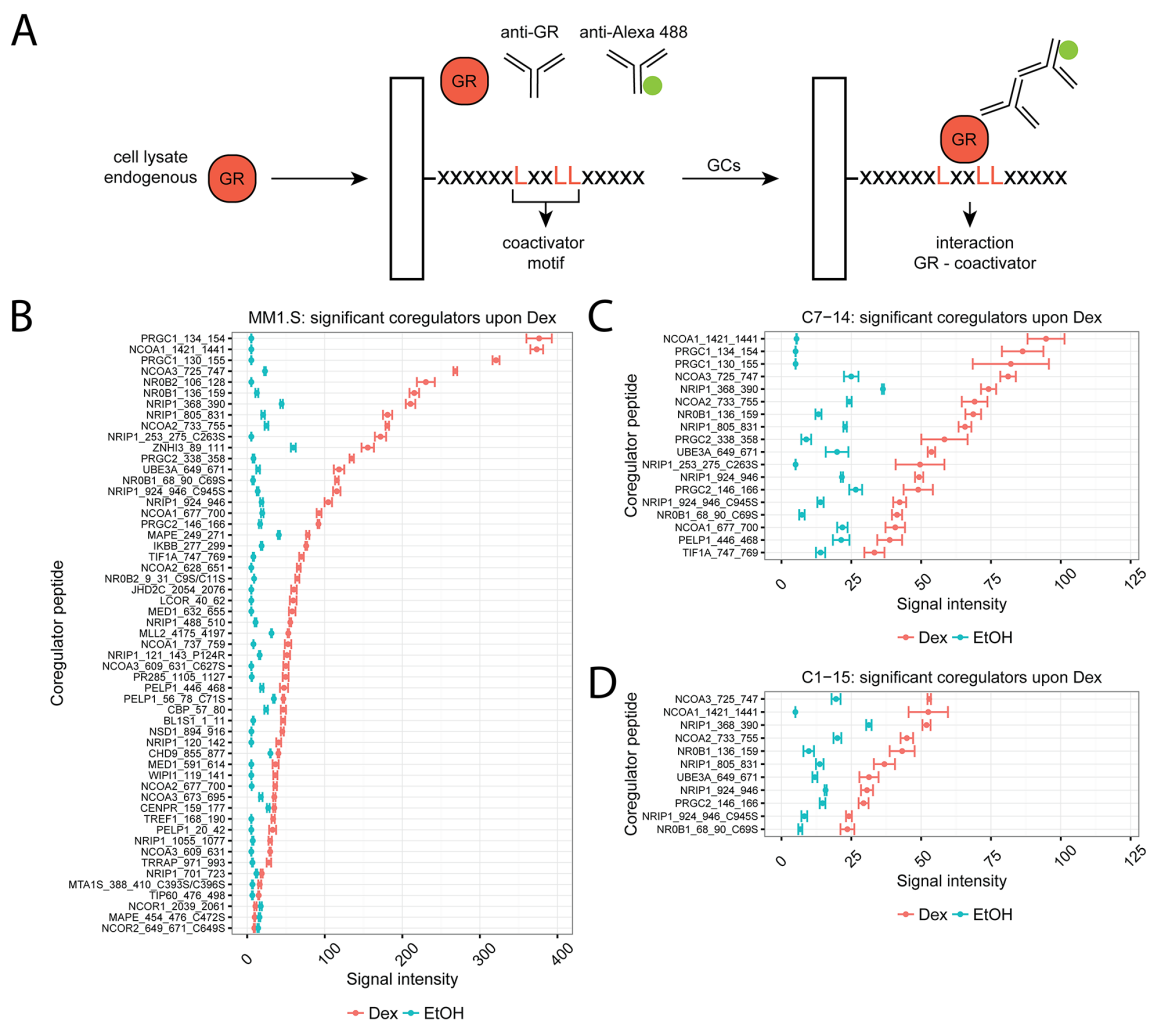
Coregulator profiling of endogenous GR in MM and ALL cells using MARCoNI **(A)** MARCoNI principle. Cell lysates containing endogenous GR and the appropriate primary (anti-GR) and secondary (anti-Alexa 488) antibodies are added onto PamChip arrays, containing spotted coregulator peptides. Interactions between a coregulator peptide and GR result in a bright spot in the readout and are quantified. **(B)** MM1.S, **(C)** C7-14 or **(D)** C1-15 cells were treated for 2h with solvent or Dex (1μM). Protein lysates were prepared and subjected to MARCoNI analyses. The plots represent the mean signal intensity +/- the standard error of the mean (SEM) of 3 biological replicates. Coregulators responding statistically significant to Dex treatment are displayed and were ranked according to Dex response. Statistical analysis was performed in R, using Welch t-tests corrected for multiple testing using the false discovery rate (FDR, 5%).

We applied the MARCoNI technology to determine endogenous GR-coregulator interactions in cell lysates. In short, cell lysate containing endogenous GR is added on the PamChip together with a primary GR and a secondary detection antibody (Figure 1A). If an interaction between, in this case, a coactivator binding motif and GR takes place, a bright spot is observed in the readout. Therefore, it is the combined presence versus absence and the intensity of the spots that will determine the NR coregulator profile.

We determined the endogenous GR coregulator profile of GC-sensitive MM and ALL cell lines, i.e. MM1.S and CEM-C7-14 (in short C7-14), respectively, and of a GC-resistant, yet GR-containing, ALL cell line, i.e. CEM-C1-15 (in short C1-15) [28, 29]. To this end, MM1.S, C7-14 and C1-15 cells were treated for 2h with solvent (EtOH) or Dex and subjected to MARCoNI analysis. Supplementary Figure 1 shows the coregulator peptides that interacted with endogenous GR in these cell lines with a mean signal intensity of more than 10 (cutoff) and ranked according to the Dex response. We further identified those coregulators that responded significantly to Dex treatment (Figure 1A-1C) and for which the corresponding p-values are listed in Table 1.

**Table 1:** Significant coregulators upon Dex treatment per cell line. A Welch t-test was used to identify coregulators that significantly respond to Dex treatment per cell line. The p-values were corrected for multiple testing using FDR (5%).

MM1.S	
coregulator	p-value
NCOA2_733_755	0.00011
IKBB_277_299	0.00014
PRGC2_146_166	0.00014
NCOA3_725_747	0.00019
NRIP1_488_510	0.00019
PRGC2_338_358	0.00115
NR0B1_68_90_C69S	0.00115
MLL2_4175_4197	0.00115
PRGC1_130_155	0.00208
NCOA1_677_700	0.00226
NR0B1_136_159	0.00297
NRIP1_805_831	0.00297
MAPE_249_271	0.00297
NCOA1_1421_1441	0.00337
NRIP1_368_390	0.00447
NCOA3_609_631	0.00488
PELP1_56_78_C71S	0.00488
TIP60_476_498	0.00488
NRIP1_924_946	0.00527
TIF1A_747_769	0.00611
NRIP1_924_946_C945S	0.00619
NCOA2_628_651	0.00639
PRGC1_134_154	0.00738
NRIP1_253_275_C263S	0.00738
UBE3A_649_671	0.00738
NSD1_894_916	0.00738
NR0B2_9_31_C9S/C11S	0.00791
NR0B2_106_128	0.00889
ZNHI3_89_111	0.00965
BL1S1_1_11	0.01144
NRIP1_1055_1077	0.01188
JHD2C_2054_2076	0.01206
NCOA3_673_695	0.01206
**coregulator**	**p-value**
CHD9_855_877	0.01206
NCOA2_677_700	0.01349
TREF1_168_190	0.01439
NCOA3_609_631_C627S	0.01492
LCOR_40_62	0.01543
MED1_632_655	0.01576
WIPI1_119_141	0.01682
PR285_1105_1127	0.01683
CBP_57_80	0.01746
NRIP1_120_142	0.01771
NCOA1_737_759	0.01812
NRIP1_121_143_P124R	0.01984
NRIP1_701_723	0.02870
NCOR2_649_671_C649S	0.02870
MED1_591_614	0.03035
TRRAP_971_993	0.03035
MAPE_454_476_C472S	0.03533
PELP1_446_468	0.03977
CENPR_159_177	0.04460
PELP1_20_42	0.04577
NCOR1_2039_2061	0.04638
MTA1S_388_410_C393S/C396S	0.04964
**C7-14**	
**coregulator**	**p-value**
NCOA3_725_747	0.00278
NR0B1_68_90_C69S	0.00467
NR0B1_136_159	0.00601
NRIP1_805_831	0.00698
NRIP1_924_946_C945S	0.00698
NRIP1_924_946	0.00698
NRIP1_368_390	0.01426
NCOA1_1421_1441	0.01496
PRGC1_134_154	0.01738
NCOA2_733_755	0.01738
UBE3A_649_671	0.01738
NCOA1_677_700	0.03047
**coregulator**	**p-value**
TIF1A_747_769	0.03216
PRGC2_338_358	0.03589
PRGC1_130_155	0.04393
NRIP1_253_275_C263S	0.04668
PRGC2_146_166	0.04668
PELP1_446_468	0.04668
**C1-15**	
**coregulator**	**p-value**
NCOA3_725_747	0.00508
NRIP1_368_390	0.00508
NRIP1_924_946_C945S	0.00508
NCOA2_733_755	0.00848
PRGC2_146_166	0.01986
NR0B1_136_159	0.03062
NRIP1_805_831	0.04223
NR0B1_68_90_C69S	0.04223
NRIP1_924_946	0.04223
NCOA1_1421_1441	0.04452
UBE3A_649_671	0.04586

MM1.S cells showed the largest number (55) differentially responding coregulators and the highest signal intensity, i.e. strongest GR-coregulator interactions, when compared to C7-14 or C1-15 (resp. 18 and 11 coregulators peptides) (Figure 1A-1C). Also, in MM1.S cells most coregulator peptides (52/55) showed increased signal intensity following Dex treatment, with PGC-1ɑ (PRGC1), SRC-1 (NCOA1), SRC-3 (NCOA3), SHP (NR0B2) and DAX-1 (NR0B1) as the coregulators with the highest signal intensities. However, for 3/55 coregulators the signal intensity of the Dex response was significantly lower compared to solvent; these were the corepressors NCOR1, NCOR2 and PRAME (or MAPE) (Figure 1A). In C7-14 cells, the signal intensities were about two-fold higher than in the GC-resistant counterpart C1-15, with SRC-1 (NCOA1), PGC-1ɑ (PRGC1), SRC-3 (NCOA3), RIP140 (NRIP1) and SRC-2 (NCOA2) displaying the highest signal intensities. Although the number of significant coregulator peptides in C7-14 (18) might seem larger than in C1-15 (11), these were mostly different peptides of the same coregulator (Figure 1C, 1D). Nevertheless, the strongest interacting coregulators in C1-15 were SRC-3 (NCOA3), SRC-1 (NCOA1), RIP140 (NRIP1), SRC-2 (NCOA2) and DAX-1 (NR0B1).

In summary, we extended the MARCoNI technology to the level of the endogenous GR in cell lysates and determined the coregulator profile of MM and ALL cell lines.

### Comparison of the GR coregulator profiles of GC-sensitive MM and ALL cells

**Figure 2:**
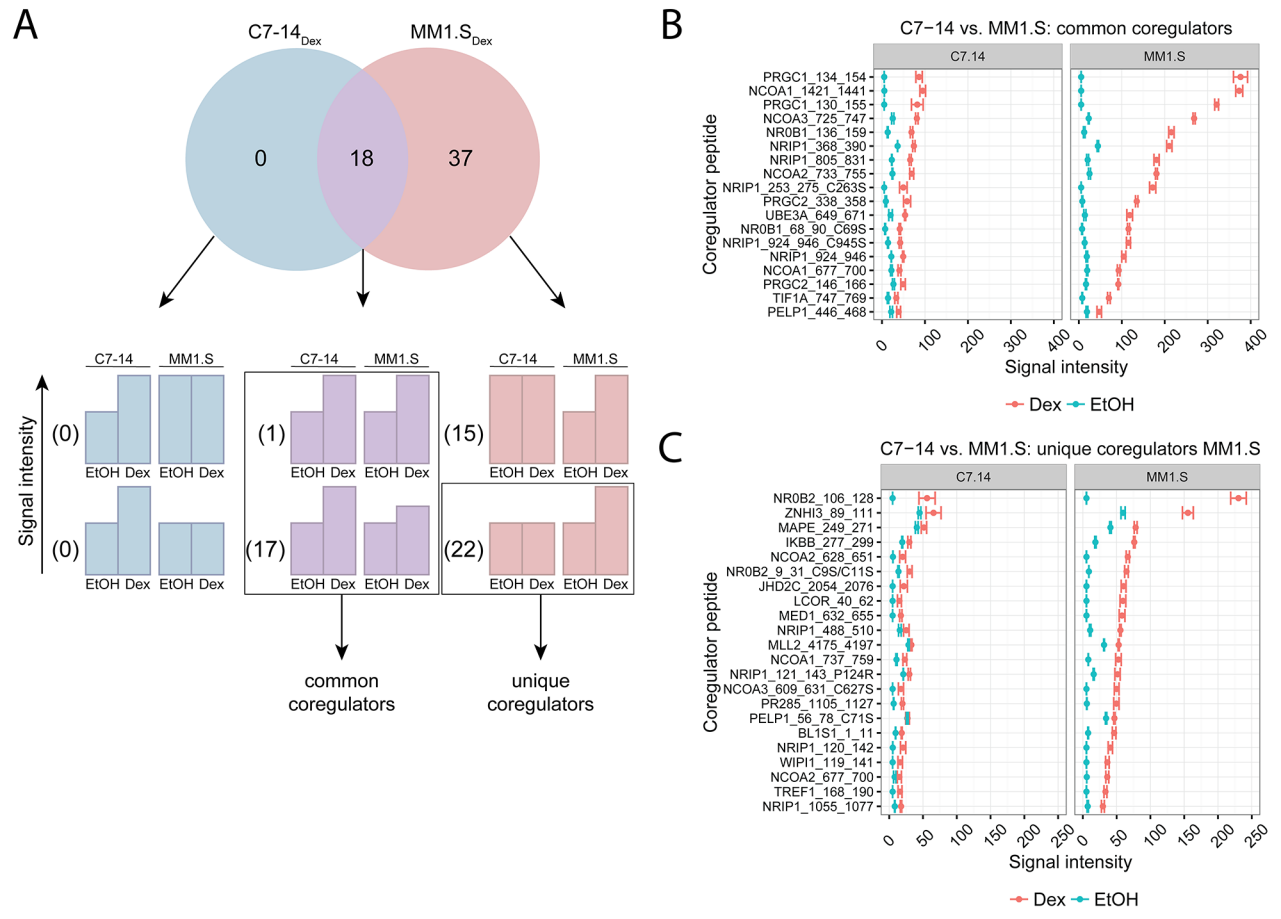
Comparing GR coregulator profiles of GC-sensitive ALL and MM cells C7-14 (ALL) and MM1.S (MM) cells were treated for 2h with solvent or Dex (1μM). Protein lysates were prepared and subjected to MARCoNI analyses. **(A)** The Venn-diagram shows the number of coregulators that respond significantly to Dex treatment in C7-14, MM1.S, or in both. For each segment of the Venn-diagram, coregulator peptides are further subdivided into 2 groups depending on whether the Dex responses were different (lower bar charts) or not (upper bar charts) between the cell lines; accompanied by the actual number of coregulators that display such a response. Coregulators defined as common or unique between C7-14 and MM1.S are indicated. **(B)** Common coregulators between C7-14 and MM1.S, **(C)** unique coregulators for MM1.S compared to C7-14. The plots represent the mean signal intensity +/- SEM of 3 biological replicates. Coregulators responding statistically significant to Dex treatment are displayed and were ranked according to Dex response in MM1.S. Statistical analysis was performed in R, using Welch t-tests corrected for multiple testing using FDR (5%).

We used MARCoNI to further characterize the similarities and differences in the endogenous GR coregulator profile of GC-sensitive MM (MM1.S) and ALL (C7-14) cells, following 2h treatment with solvent (EtOH) or Dex. The Venn-diagram (Figure 2A) displays the number of significantly responding coregulator peptides upon Dex treatment when comparing MM1.S with C7-14 cells. We found 0 coregulator peptides in C7-14 alone (C7-14_Dex_), 37 in MM1.S alone (MM1.S_Dex_) and 18 in both C7-14 and MM1.S (intersect) (Figure 2A). We further subdivided the coregulator peptides of each segment of the diagram into 2 groups. One group (upper bar charts) contains the coregulator peptides for which the Dex response is not different between the cell lines of the comparison. The second group (lower bar charts) contains the coregulator peptides with a differential Dex response between the cell lines (Figure 2A).

Zooming in on the intersect, there was 1 coregulator peptide for which the Dex responses between MM1.S and C7-14 were not different and 17 coregulator peptides with differential Dex response between the cell lines (Figure 2A). Together, these 18 coregulator peptides were termed common between MM1.S and C7-14 and the corresponding signal intensities are depicted in Figure 2B. For instance, for PRGC1_134_154 the signal intensity in MM1.S was about four-fold higher than in C7-14 (Figure 2B). Moreover, the coregulators with the highest signal intensities in MM1.S and C7-14 were PGC-1ɑ (PRGC1), SRC-1 (NCOA1), SRC-3 (NCOA3), DAX-1 (NR0B1) and RIP140 (NRIP1).

The MM1.S_Dex_ segment showed 15 coregulator peptides with a Dex response that was not different from the Dex response in C7-14, while there were 22 coregulator peptides with a differential Dex response between the cell lines (Figure 2A). Therefore, only the latter 22 coregulator peptides could be termed unique for MM1.S when compared to C7-14 and their signal intensities are presented in Figure 2C. Moreover, the list of unique coregulator peptides of the first cell line of the comparison must always be verified with the list of significant coregulator peptides in the second cell line. If a coregulator peptide is present in both lists, or different coregulator peptides for the same coregulator are present in both lists, then the corresponding coregulator cannot be designated unique in one cell line. For instance, NR0B2_106_128 (Figure 2C) was listed as unique in MM1.S, but still showed a considerable, but not significant, Dex response in C7-14 (Figure 2C). In this case, the statistical procedure did not distinguish between no significant response upon treatment and no significant response due to large SEM. In addition, though NCOA2_628_651 was listed as a unique coregulator peptide in MM1.S, it is only one out of four coregulator peptides that represent SRC-2 (NCOA2) (Figure 2C). Since another SRC-2 coregulator peptide, i.e. NCOA2_733_755, was present in the list of significant coregulators in C7-14 (Table 1), SRC-2 cannot be truly considered as a unique coregulator in MM1.S. Therefore, the top five coregulators that are truly unique in MM1.S were TRIP3 (ZNHI3), PRAME (MAPE), NFKBIB (IKBB), JMJDAC (JHD2C) and LCOR (Figure 2C).

### Comparison of the GR coregulator profiles of GC-sensitive and GC-resistant ALL cells

**Figure 3:**
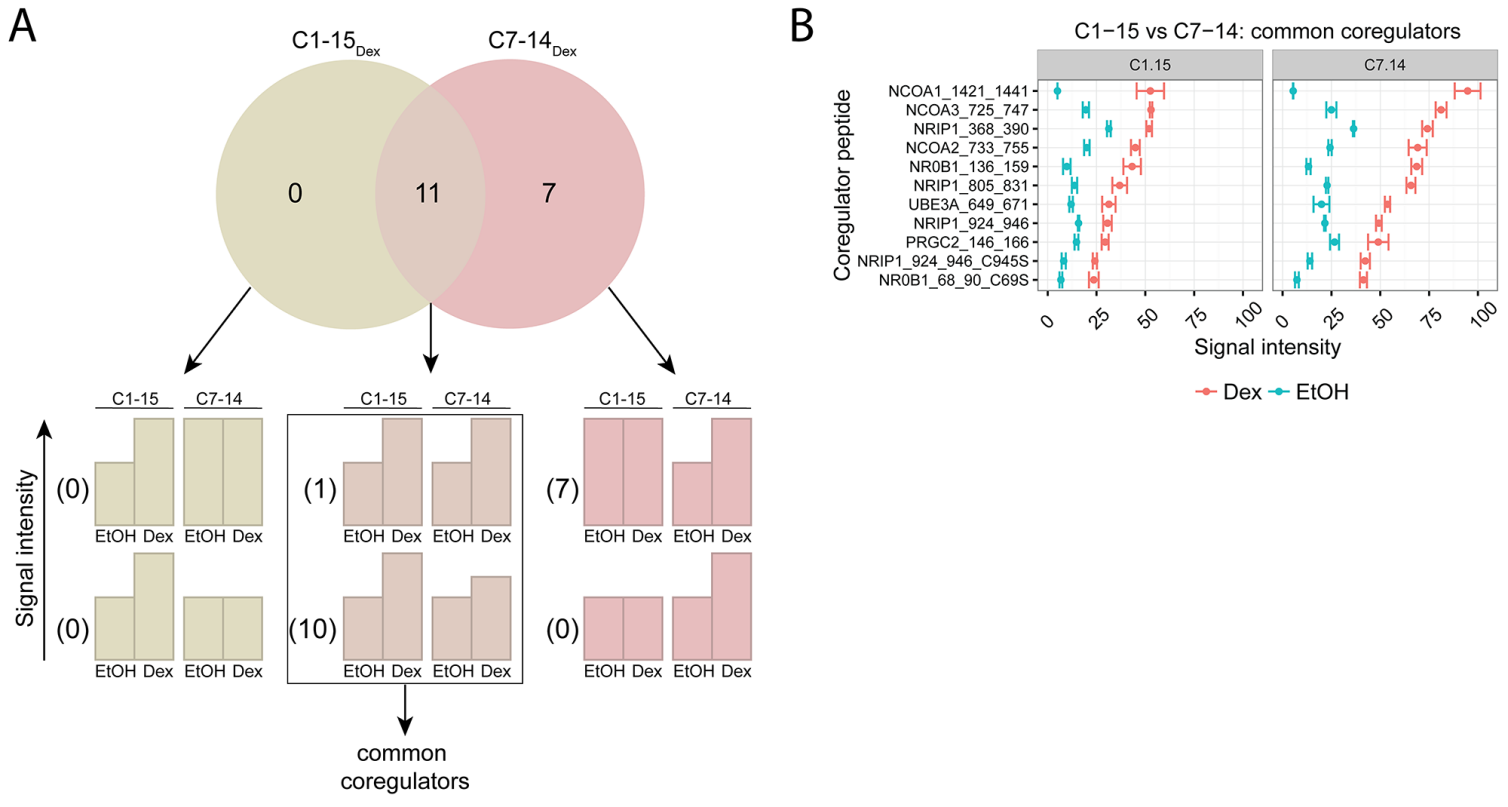
Comparing GR coregulator profiles of GC-sensitive and -resistant ALL cells C1-15 (GC-resistant) and C7-14 (GC-sensitive) cells were treated for 2h with solvent or Dex (1μM). Protein lysates were prepared and subjected to MARCoNI analyses. **(A)** The Venn-diagram shows the number of coregulators that respond significantly to Dex treatment in C1-15, C7-14, or in both. For each segment of the Venn-diagram, coregulator peptides are further subdivided into 2 groups depending on whether the Dex responses were different (lower bar charts) or not (upper bar charts) between the cell lines; accompanied by the actual number of coregulators that display such a response. Coregulators defined as common between C1-15 and C7-14 are indicated. **(B)** Common coregulators between C1-15 and C7-14. The plot represents the mean signal intensity +/- SEM of 3 biological replicates. Coregulators responding statistically significant to Dex treatment are displayed and were ranked according to Dex response in C7-14. Statistical analysis was performed in R, using Welch t-tests corrected for multiple testing using FDR (5%).

To identify common and unique coregulators of endogenous GR between GC-sensitive (C7-14) and GC-resistant (C1-15) ALL cell lines, we treated these cells for 2h with solvent or Dex and applied the MARCoNI technology. The number of coregulator peptides that were significantly triggered upon Dex (Figure 3A) was: 0 in C1-15 alone (C1-15_Dex_), 7 in C7-14 alone (C7-14_Dex_) and 11 in both C1-15 and C7-14 (intersect).

More in detail, in the intersect segment 10 coregulator peptides did and 1 did not show a differential Dex response between the cell lines. Together these 11 coregulator peptides were considered as common between C7-14 and C1-15 and their signal intensities are displayed in Figure 3B. Interestingly, the signal intensities in the GC-sensitive C7-14 cells were consistently about two-fold higher than in the GC-resistant C1-15 cells. For these cell lines, the coregulators with the highest signal intensities were: SRC-1 (NCOA1), SRC-3 (NCOA3), RIP140 (NRIP1), SRC-2 (NCOA2) and DAX-1 (NR0B1).

Remarkably, we did not identify coregulators that were unique for either C1-15 or C7-14. For C7-14 we did find 7 coregulator peptides that respond significantly to Dex treatment, but the Dex response in C7-14 was not different from the Dex response in C1-15 and thus these coregulator peptides cannot be defined as truly unique for C7-14.

### Comparison of the GR coregulator profiles of MM and lung carcinoma cells

We also compared the endogenous GR coregulator profile of a lymphoid malignancy (MM, e.g. MM1.S) with a solid cancer (lung carcinoma, e.g. A549), following 2h stimulation with solvent or Dex. Supplementary Figure 2 shows the coregulator peptides that are significantly different upon Dex treatment in A549 cells, together with their corresponding p-values (Supplementary Table 1). In A549 cells, RIP140 (NRIP1), SRC-1 (NCOA1), PGC-1ɑ (PRGC1), TRIP-3, (ZNHI3) and SRC-3 (NCOA3) were the coregulators with the highest signal intensity. Remarkably, 8 coregulator peptides, for instance NCOR1_2039_2061, showed a significant drop in signal intensity upon Dex treatment.

**Figure 4:**
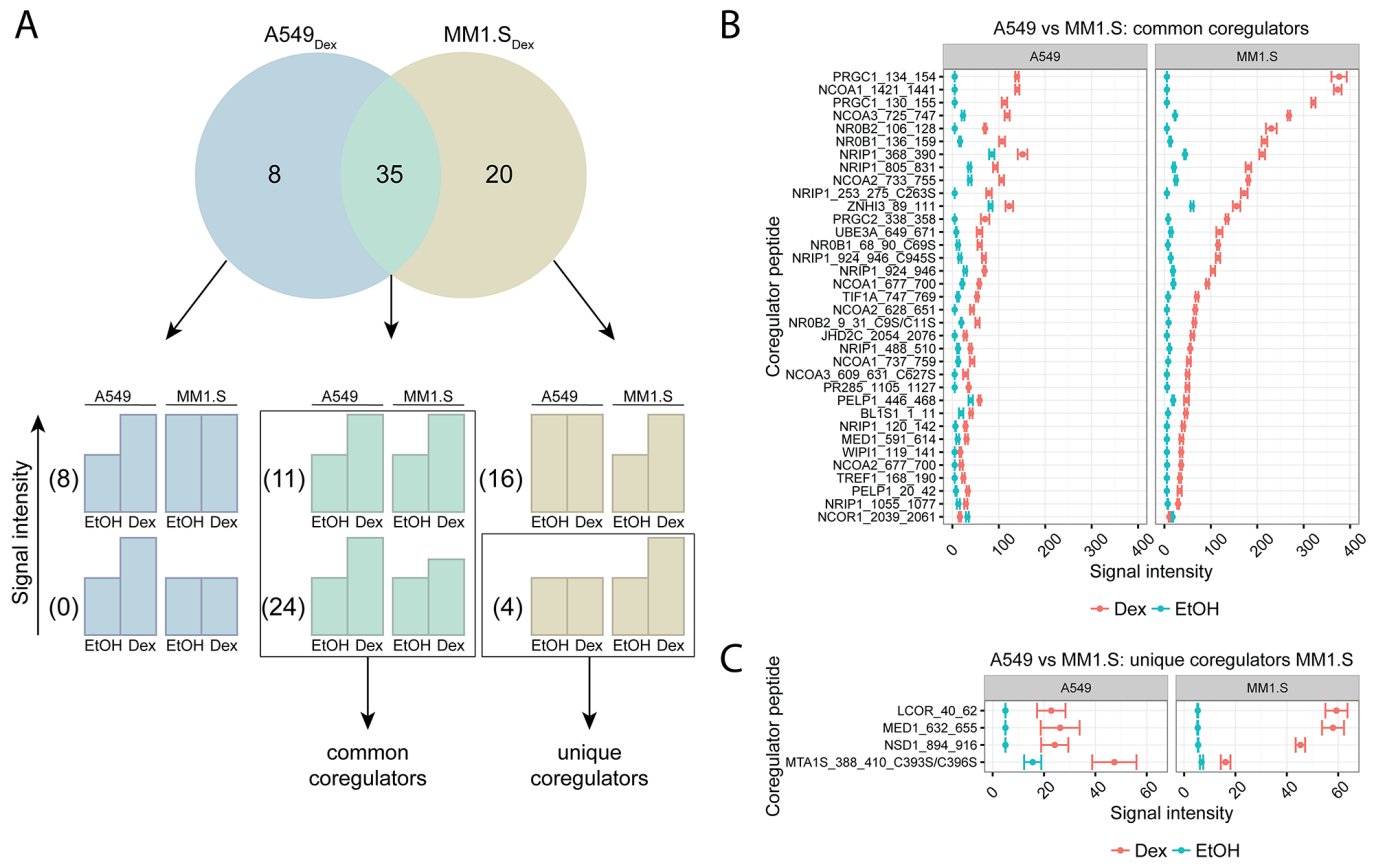
Comparing GR coregulator profiles of lung carcinoma and MM cells A549 (lung carcinoma) and MM1.S (MM) cells were treated for 2h with solvent or Dex (1μM). Protein lysates were prepared and subjected to MARCoNI analyses. **(A)** The Venn-diagram shows the number of coregulators that respond significantly to Dex treatment in A549, MM1.S, or in both. For each segment of the Venn-diagram, coregulator peptides are further subdivided into 2 groups depending on whether the Dex responses were different (lower bar charts) or not (upper bar charts) between the cell lines; accompanied by the actual number of coregulators that display such a response. Coregulators defined as common or unique between A549 and MM1.S are indicated. **(B)** Common coregulators between A549 and MM1.S, **(C)** unique coregulators for MM1.S compared to A549. The plot represents the mean signal intensity +/- SEM of 3 biological replicates. Coregulators responding statistically significant to Dex treatment are displayed and were ranked according to Dex response in MM1.S. Statistical analysis was performed in R, using Welch t-tests corrected for multiple testing using FDR (5%).

Figure 4A displays the number of coregulator peptides that significantly responded to Dex treatment: 8 in A549 alone (A549_Dex_), 20 in MM1.S alone (MM1.S_Dex_) and 35 in both A549 and MM1.S (intersect). In the intersect, 11 coregulator peptides did not and 24 did display a differential Dex response between the cell lines. In total, these 35 coregulators peptides were in common between A549 and MM1.S cells and their corresponding signal intensities are shown in Figure 4B. Overall, the signal intensities in the MM1.S cells were up to three-fold higher than in A549 cells. The top five common coregulators between these cell lines are PGC-1ɑ (PRGC1), SRC-1 (NCOA1), SRC-3 (NCOA3), SHP (NR0B2), DAX-1 (NR0B1).

No unique coregulators could be identified in A549 cells compared to MM1.S cells. Yet, 8 coregulator peptides were differentially regulated upon Dex treatment in A549 cells and not in MM1.S cells, but their corresponding Dex responses did not differ and thus these could not be defined as unique. In MM1.S cells, 4 unique coregulator peptides were found and their signal intensities are shown in Figure 4C. However, these 4 coregulator peptides still show a considerable Dex response in A549 cells (Figure 4C). In this case, the statistical procedure could not differentiate between no significant response upon treatment and no significant response due to large SEM.

### Validation of endogenous GR-SRC-1 interaction and correlation with GR protein levels

**Figure 5:**
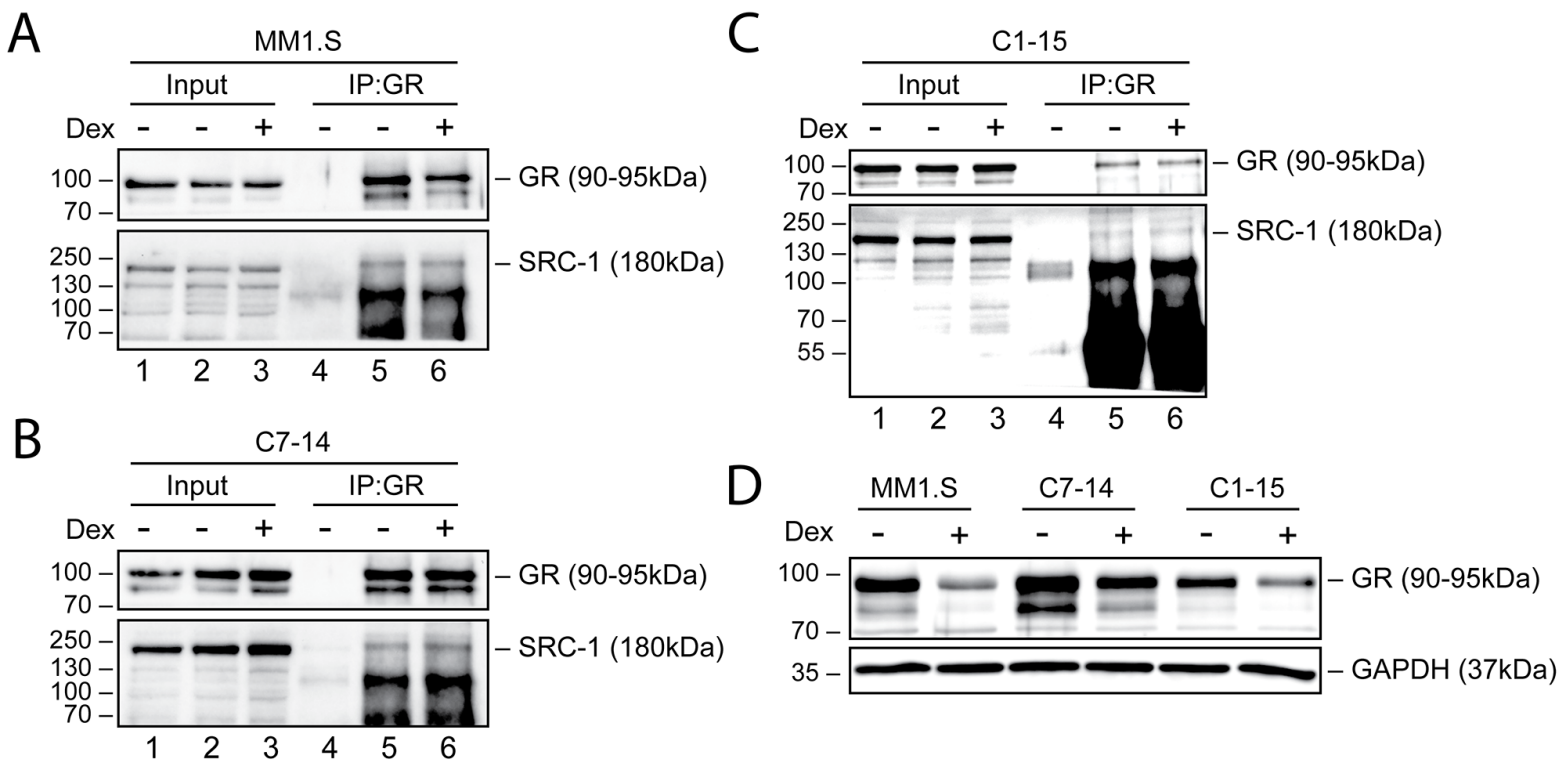
Endogenous GR levels and interaction with SRC-1 in MM and ALL cells **(A)** MM1.S, **(B)** C7-14, **(C)** C1-15 cells or **(D)** MM1.S, C7-14 and C1-15 cells were treated for 2h with solvent or Dex (1μM). Protein lysates, (A-C) NP-40-based or (D) M-PER-based, were prepared and endogenous GR co-immunoprecipitation (A-C) was performed, followed by WB analysis to detect the protein levels of GR (90-95kDa) or SRC-1 (180kDa), with GAPDH (37kDa) serving as loading control. As a negative control for IP, a non-specific antibody was used (lane 4). WB results are representative for 2 (A-C) or 3 (D) independent experiments.

To validate the MARCoNI results, we determined whether GR interacts with SRC-1 at the endogenous level in MM1.S, C7-14 and C1-15 cells, as SRC-1 appeared as one of the strongest interacting coregulators of GR in all cell line comparisons. To this end, we treated MM1.S, C7-14 and C1-15 cells for 2h with solvent or Dex, immunoprecipitated GR and assayed its interaction with SRC-1 via Western blot (WB) analysis. Figure 5A-5C shows that immunoprecipitation of GR was successful in all cell lines, as evidenced by the presence of a GR band in the IP fraction (lanes 5 and 6), and its absence in lane 4 (aspecific antibody control). Supplementary Figure 3A-3C includes the HEK293T positive control for the SRC-1 antibody, which confirmed its specificity and indicated that the SRC-1 band is located at 180kDa. Moreover, SRC-1 was present in the immunoprecipitation (IP) fraction of all cell lines, confirming the interaction between SRC-1 and GR (Figure 5A-5C, lanes 5 and 6). In MM1.S and C1-15 cells, Dex treatment did not alter the SRC-1 levels in the IP fraction, while in C7-14 cells the levels of SRC-1 were slightly increased upon Dex treatment (Figure 5A-C).

We also wondered whether GR protein levels in MM1.S, C7-14 and C1-15 corresponded to the observed signal intensities in the MARCoNI assay. Therefore, we determined GR protein levels in these cell lines after 2h treatment with solvent or Dex and quantified these by densitometric analysis (Supplementary Figure 4A). In the absence of ligand, GR protein levels were the highest in C7-14, then in MM1.S and the lowest in C1-15 (Figure 5D). Upon Dex stimulation, GR levels underwent homologous downregulation, albeit to a varying degree that depended on the cell line. MM1.S displayed the lowest GR levels upon Dex treatment, although these cells showed the highest MARCoNI signal intensity. GC-sensitive C7-14 cells did not only exhibit higher GR protein levels than GC-resistant C1-15 cells in response to Dex, but also displayed the strongest signal for an additional GR isoform around 80kDa.

Summarized, our results confirmed the interaction between endogenous GR and SRC-1 in MM1.S, C7-14 and C1-15 cells and suggested that GR protein levels do not correlate with the signal intensity of the MARCoNI assay.

### Endogenous GR levels and coregulator profile of PBMCs

**Figure 6:**
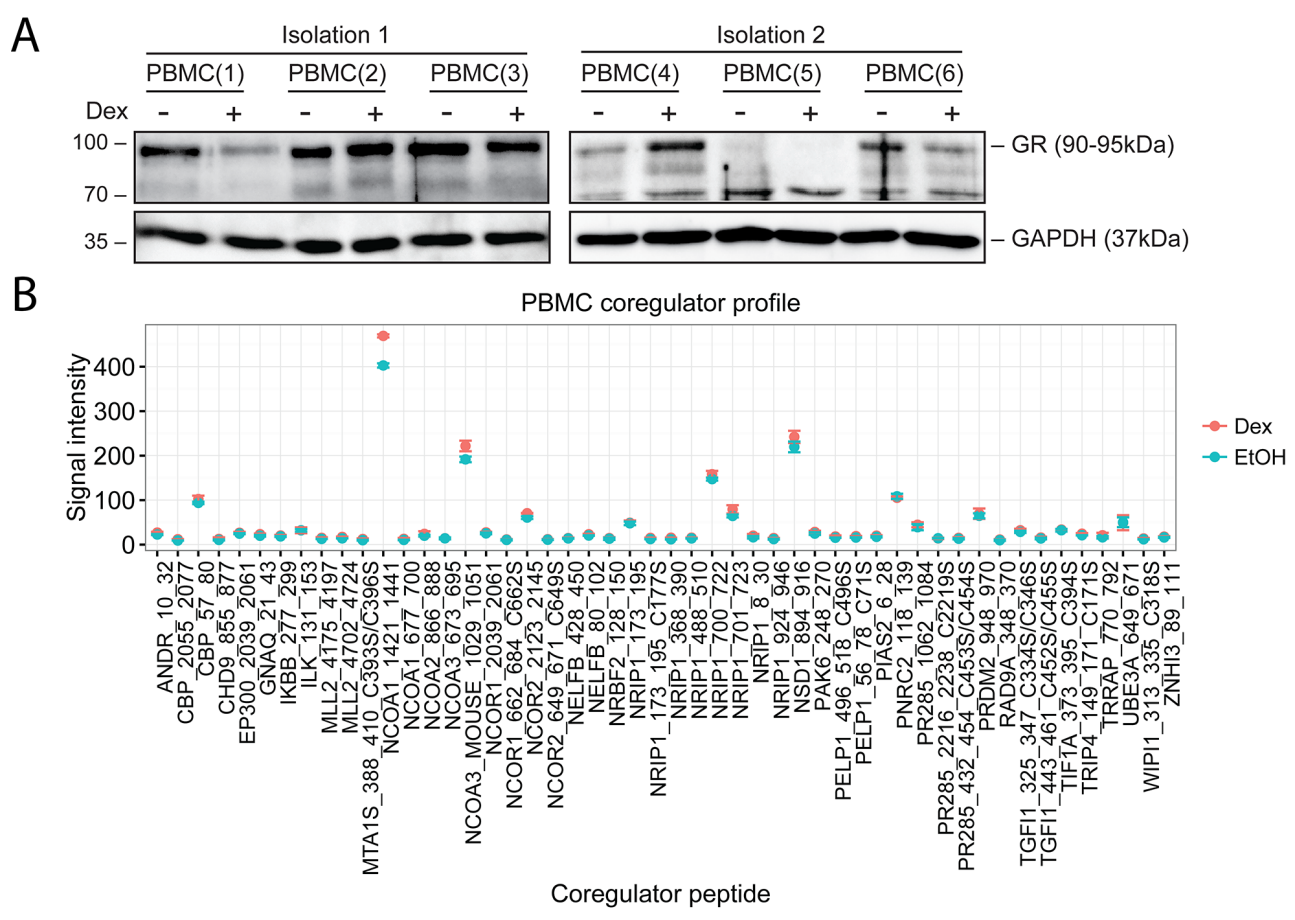
Endogenous GR levels and coregulator profile of PBMCs PBMCs were isolated and treated for 2h with solvent or Dex (1μM). Protein lysates were prepared and subjected to **(A)** WB or **(B)** MARCoNI analyses. (A) WB analysis was performed to detect the protein levels of GR (90-95kDa), GAPDH (37kDa) served as loading control. WB results represent two independent PBMC isolations of each time 3 biological replicates. (B) The coregulator plot represents the mean signal intensity +/- SEM of 3 biological replicates. Data analysis was performed in R.

As a step towards patient samples, we isolated PBMCs from healthy volunteers, assayed GR protein levels, quantified these by densitometric analysis (Supplementary Figure 4B) and determined the corresponding coregulator profile after 2h stimulation with solvent or Dex. Figure 6A shows that upon Dex treatment, GR protein levels were either downregulated, unaltered, or even increased, indicating that GR protein levels in PBMCs are quite variable. Figure 6B demonstrates that Dex treatment did not differentially affect the signal intensity of the coregulator peptides compared to solvent. This is conceivable since endogenous cortisol that is present in blood can influence the signal intensity. In this case, the solvent control cannot be considered as a true negative control due to the endogenous cortisol levels. Nevertheless, coregulator peptides with signal intensities above 50 could still be considered as interactors of endogenous GR. The top five coregulators in PBMCs are therefore: SRC-1 (NCOA1), H3-K36-HMT (NSD1), SRC-3 (NCOA3), RIP140 (NRIP1) and PNRC2.

## DISCUSSION

In this report, we determined the coregulator profile of endogenous GR in cell lysates of MM, ALL, lung carcinoma cell line models and PBMCs. The interaction profile of coregulators with NRs is defined by different factors. The origin of both the ligand, i.e. synthetic or endogenous, and the NR, i.e. endogenous, overexpressed or recombinant, affects the NR conformation, PTM-status and ultimately the recruitment of certain coregulators [26, 30]. Desmet *et al.* recently showed that the combination of Dex with the selective GR modulator compound A (CpdA) gave rise to altered GR coregulator recruitment. NR0B2 (SHP) interacted more strongly with GR upon Dex/CpdA combination compared to Dex alone [31]. Vice versa, the recruited coregulators that interact with the NR can influence the NR conformation and PTM-status and undergo PTMs themselves. For instance, SMRT (NCOR2) phosphorylation by ERK2 was shown to disrupt the SMRT-corepressor complex and inhibited transcriptional repression [32]. Also, the cell type specific expression and competition between coregulators co-determines the NR-coregulator interaction profile. Altogether, these factors substantiate the reason why so far more than 400 coregulators have been identified [5, 26].

We extended the MARCoNI technology to monitor coregulator associations with endogenous GR in cell lysates [26]. So far, coregulator profiling from cell and tumor lysates was only reported for ERɑ [27]. The major advantage of this technology is that it allows the simultaneous detection of up to 67 coregulator-NR interactions, yet, there are also limitations. For instance, there can be competition between the coregulator peptides and endogenous coregulators present in the cell lysate for binding the NR, which can mask interactions on the PamChip [26]. Given that coregulators are expressed in a cell-type specific manner, certain interactions between a coregulator peptide and the NR could also be artificial if the coregulator is not endogenously present in the cell lysate. Moreover, neither coregulator PTMs nor the promoter context that influences NR conformation and coregulator recruitment [3], nor other TFs that bind to target gene promoters and their affiliate coregulators are considered by this technology. Nevertheless, the MARCoNI technology has proven to be one of the only tools that permits simultaneous screening of a vast number of coregulator-NR interactions [31, 33, 34]. Moreover, MARCoNI may prove useful to dissect coregulator profile changes in a pathological setting, given that coregulators are master regulators of diseases [2].

Using MARCoNI, we identified SRC-1/2/3, PGC-1ɑ, RIP140 and DAX-1 as the strongest interacting coregulators of MM and ALL cells. The highest MARCoNI signal intensities were found in MM1.S cells compared to C7-14 and C1-15 cells. It is conceivable that the number of GR units (not total GR content) that interacts with a certain coregulator peptide to produce a signal is different between cell lines, resulting in different signal intensities between cell lines. We speculate that the affinity of endogenous GR for coregulators may be higher in MM1.S cells, compared to the other cell lines, indicating that the affinity of NRs for coregulators may well be cell-type specific. In addition, the PTM-status of the NR can influence the obtained MARCoNI signal intensity. Indeed, for ERɑ it was shown that increased ERɑ Ser305 phosphorylation induced increased coregulator binding [27]. Moreover, GR Ser211 phosphorylation was shown to increase transcriptional activity by promoting a conformation change that facilitates coregulator binding [35]. In this context, Lynch and co-workers compared GR Ser211 and Ser226 phosphorylation in C7-14 and C1-15 cells and found that GR Ser211 phosphorylation was predominant in C7-14 cells, while for C1-15 cells this was GR Ser226 phosphorylation [36]. This difference might serve to explain the higher MARCoNI signal intensities in C7-14 cells versus C1-15 cells.

Moreover, most coregulators that responded significantly to Dex treatment in MM1.S, C7-14 or C1-15 cells displayed increased signal intensity, which means that an interaction between GR and a certain coregulator takes place upon ligand induction. However, in a few cases the signal intensity dropped upon Dex stimulation. It is conceivable that in the absence of ligand a proportion of coregulators are constitutively bound to the NR. Upon NR stimulation, these interactions are lost and would result in decreased signal intensity in the MARCoNI assay. For instance, we observed this response for the corepressors NCOR1 and NCOR2 in MM1.S and A549 cells, but also for e.g. the coactivator CBP in A549 cells. The latter exemplifies the concept that coactivators can act as corepressors and vice versa [3]. In this context, the group of Rogatsky showed that the coactivator GRIP1 (SRC-2) facilitated GC-induced anti-inflammatory actions *in vivo* by acting as a corepressor [5]. Also MTA1, a known corepressor for ERɑ, was shown to function as a coactivator for the gene *BCAS3* that was described to be overexpressed and amplified in breast cancer [37].

We showed that GC-sensitive MM1.S compared to C7-14 cells display a more dynamic coregulator profile. We propose that this arises from a more GC-responsive GR in MM1.S cells compared to C7-14 cells, which would be also conformationally more flexible to interact more strongly and with a wider range of coregulators. The group of Yamamoto recently showed that GR plasticity is an important factor in the regulation of transcription [30], supporting our hypothesis. This is in line with why unique coregulators could only be identified in MM1.S. Yet, in MM1.S we also identified 15 coregulator peptides that responded to Dex treatment, but for which the Dex response was not different from the one in C7-14. We suggest that these coregulator peptides represent coregulators that are constitutively bound to GR.

Moreover, we found that PGC-1ɑ, SRC-1/3, DAX-1 and RIP140 were the coregulators with the highest signal intensities between MM and ALL cell lines. Up to now, the role of coregulators in hematological malignancies is not well characterized. In MM, STAT3 was shown to recruit CBP/p300 coactivators for the transactivation of its target genes, a process in which SRC-1 is generally not required, and thereby promotes growth and inhibits apoptosis of MM cells [38, 39]. Negative modulation of STAT3 by peroxisome proliferator activated receptor γ (PPARγ) and ER was shown in MM via a direct mechanism or by recruitment of the coregulators NCOR2 or PIAS [38]. Moreover, in acute myeloid leukemia (AML) the HAT monocytic leukemia zinc finger protein (MOZ) was shown to generate fusion proteins with the coactivators p300, CBP and SRC-2 via chromosomal translocations [40]. These MOZ fusion genes deregulate MOZ-controlled target gene transcription and thereby repress differentiation, induce hyperproliferation and disturb normal hematopoiesis [40]. Also, in chronic lymphoid leukemia (CLL), transcriptional profiling of chromosome 2p gain CLL cells identified, amongst others, SRC-1 to be significantly upregulated in these cells [41]. In contrast, in B-cell lymphoma the presence of SRC-3 was shown to have anti-proliferative effects [42]. This suggests that the SRC coregulator family may have opposing effects in lymphoid malignancies, i.e. pro-proliferative versus anti-proliferative, depending on the cell type. In addition, Millard *et al.* suggest that the SMRT/NCOR complex, containing HDAC3, might be a key target in diseases for which pan-HDAC inhibitors have proven their use [43]. MM serves as an example, as the pan-HDAC inhibitor panobinostat has been approved for the treatment of relapsed/refractory MM patients who had two prior lines of treatment [44] and since HDAC3 was recently identified as a novel therapeutic target in MM as HDAC3 inhibitors were shown to induce MM cell death [45].

We identified SRC-1 (NCOA1), SRC-3 (NCOA3), RIP140 (NRIP1), SRC-2 (NCOA2) and DAX-1 (NR0B1) as the strongest interacting coregulators of endogenous GR in GC-sensitive C7-14 and GC-resistant C1-15 cells. The MARCoNI signal intensities in the C7-14 cells were vastly higher than in C1-15 cells, suggesting that the affinity of GR for coregulators may be higher in C7-14 than in C1-15 cells. Moreover, since GR is more GC-responsive in C7-14 cells compared C1-15 cells, it also explains why 7 coregulator peptides respond to Dex exclusively in C7-14 cells compared to 0 in C1-15 cells. We propose that GR is more flexible in C7-14 cells to interact more strongly and with more coregulators, while GR seems more rigid to do so in C1-15 cells. However, we did not find unique coregulators for either cell line, indicating that GC resistance in C1-15 cells cannot be explained by qualitative but more likely (partly) by quantitative differences in the coregulator profile, possibly also arising from a more rigid GR in C1-15 cells. Coregulators have been implicated in GC and therapy resistance before. GC-induced GR gene repression was shown to be conveyed by blocking transcription initiation via the formation of a long-range interaction between an NCOR1-containing complex at the transcription start site and an intragenic negative GC response element (nGRE). In this sense, long-term GC treatments could lead to constitutive GR gene repression and by extension GC resistance [25]. In breast cancer, SRC-3 overexpression has been linked to resistance to therapy [46–48]. Moreover, patients with high SRC-3 and HER-2 expression levels showed worse outcomes with antiestrogen therapy compared to all other breast cancer patients together [46]. In addition, a mutation in ERɑ was shown to cause constitutively activation of ERɑ by recruiting coactivators in the absence of ligand and caused resistance to antiestrogen therapy by altering conformational changes in the ERɑ ligand binding domain [48].

We found PGC-1ɑ, SRC-1, SRC-3, SHP and DAX-1 as the top five common coregulators upon comparison of MM (MM1.S) cells with lung carcinoma (A549) cells. Although we found unique coregulator peptides for MM1.S cells, these coregulators still displayed a considerable Dex response, and were considered as false positives. In lung carcinoma cells, we did not identify coregulators that are uniquely expressed in these cells. Cai *et al.* showed that SRC-3 was found to be overexpressed in 27% of non-small cell lung carcinomas and was associated with rapid progression of the disease [2, 6, 49]. Besides in lung and breast cancer, coregulators have been implicated in other solid malignancies, such as prostate, endometrial and ovarian cancer, and in melanoma [2, 50]. For instance, in prostate cancer miR137 was show to suppress androgen signaling by modulating the expression of a range of coregulators including SRC-2 [51]. Moreover, Dasgupta and colleagues found that SRC-2 inhibition in mice strongly attenuates prostate cancer cell growth, survival and metastasis [52].

We confirmed the interaction of GR with SRC-1 by endogenous co-immunoprecipitation, albeit a ligand-dependent effect was largely lacking. An increase in SRC-1 protein levels upon Dex treatment was only observed in C7-14 cells. This may reflect differences in how interactions are probed by IP/WB (static) compared to MARCoNI (dynamic, real-time). Nevertheless, this suggests that co-immunoprecipitation is a suitable strategy to qualitatively rather than quantitatively validate coregulator-NR interactions. In addition, we found that GR protein levels do not seem to correlate with the MARCoNI signal intensity. Indeed, MM1.S cells showed the highest signal intensities compared to C7-14 and C1-15 cells, but showed the lowest GR protein levels upon Dex treatment. This confirms that not GR protein levels, but the affinity of GR for the coregulators determines the MARCoNI signal intensity and thus their interaction strength. Moreover, we realize that GR levels responded differently to Dex treatment in Figure 5A-5C compared to Figure 5D, although the same induction time was used. These differences seem to arise from the use of different lysis buffers, since this was the major difference between these experiments.

We found that GR protein levels varied in PBMCs depending on the healthy volunteer from which the PBMCs were derived. In addition, we found that GC stimulation of PBMCs (mostly) did not alter the coregulator signal intensities compared to solvent. It is conceivable that some individuals may have had higher endogenous cortisol levels in their blood at the time of blood sampling, which is a stressful event. A rise in cortisol levels could further influence both GR protein levels and limit the response to synthetic GCs, as GR was probably saturated with endogenous cortisol. Obtaining a proper negative control in this context is technically challenging due to circadian and ultradian GC secretion with even inter-person variability [53]. Nevertheless, coregulator peptides with signal intensities above 50 were considered as interaction partners of GR, as the signal intensities of the cell line solvent conditions were never higher than 50. Therefore, SRC-1 (NCOA1), H3-K36-HMT (NSD1), SRC-3 (NCOA3), RIP140 (NRIP1) and PNRC2 were identified as the strongest interacting coregulators of endogenous GR in PBMCs as they displayed the highest signal intensities. Although, we do have to remark that the coregulator peptides that respond to Dex treatment in case of SRC-3 and RIP140 are different in PBMCs compared to those in MM/ALL cells. This might be explained by different conformational changes in GR, that depend on the ligand, i.e. Dex in MM/ALL and cortisol in PBMCs. Since we have shown that coregulator profiling of PBMCs is technically possible, it can be considered that coregulator profiling of MM/ALL cells from patient samples is also a feasible goal. It would also be interesting in the future to even compare PBMCs of actual patients with MM/ALL to learn whether they would respond different from what is now observed in healthy volunteers. In extension, coregulator profiling could be even used for monitoring response to therapy in patients over time.

SRCs have been implicated in proliferation, survival and metastasis of cancer cells and in therapy resistance, and thus represent key targets for the development of novel anti-cancer drugs [50]. In contrast to targeted chemotherapeutics, which block one signaling pathway, SRC-directed drugs would simultaneously target multiple pathways and could overcome aspects of acquired resistance mechanisms [54]. However, their development is hampered by the fact that SRCs lack a high affinity ligand binding domain as well as a defined enzymatic activation surface [7]. Nevertheless, the high-throughput screening of a chemical library identified the structurally unrelated bufalin and verrucarin A as SMI inhibiting the transcriptional activity of SRCs [7]. Although bufalin directly binds and degrades SRC-3 and SRC-1 via the proteasome and blocks cancer cell growth *in vitro* and *in vivo*, verrucarin selectively degrades SRC-3 without physically interacting with it and blocks cancer cell proliferation and migration [7, 55, 56]. Recently, also SI-2 was identified as a highly promising SMI of SRC-3 that selectively reduces both its transcriptional activity and concentration and potently reduces BC cell viability [57]. In contrast to SMIs, also small molecule stimulators (SMSs), such as MCB-613, have been developed. MCB-613 hyperstimulates the transcriptional activity of SRCs, thereby overloading the stress response of the cancer cells which ultimately kills them [58]. Since the SRC’s are also prominently activated by GCs in MM and ALL cells, we suggest therapeutic exploration of SRC SMIs and SMSs in hematological malignancies.

## MATERIALS AND METHODS

### Cell lines and reagents

Human multiple myeloma cells (MM1.S) and acute lymphoblastic leukemia cells (CEM-C7-14 and CEM-C1-15) were cultured in RPMI1640 glutamax (Gibco, life technologies) supplemented with 10% fetal calf serum (Greiner bio-one), 100U/mL penicillin and 0.1mg/mL streptomycin (Gibco, life technologies), and were grown in a 5% CO_2_ incubator at 37°C. MM1.S cells were purchased from ATCC, CEM-C7-14 and CEM-C1-15 cells were kind gifts from Prof. Brad E. Thompson (University of Texas Medical Branch). Human embryonic kidney cells (HEK293T) and human lung carcinoma cells (A549) were cultured in DMEM (Gibco, life technologies) supplemented with 10% fetal calf serum (Greiner bio-one), 100U/mL penicillin and 0.1mg/mL streptomycin (Gibco, life technologies), and were grown in a 5% CO_2_ incubator at 37°C. HEK293T and A549 cells were obtained from the nuclear receptor lab (NRL) (VIB-UGent). All experiments were performed using charcoal-stripped serum (Gibco, life technologies) to eliminate the influence of endogenous hormones present in fetal calf serum. All cell lines were regularly tested for mycoplasma contamination and were negative.

The glucocorticoid dexamethasone (Dex) was purchased from Sigma Aldrich and dissolved in EtOH. In all experiments the total solvent concentration was kept equal in each condition.

### Protein lysates and Western blotting (WB)

Cells were induced as indicated in the figure legends. Protein lysates were prepared by: 1) Totex lysis buffer (Hepes/KOH pH=7.9 20mM, NaCl 350mM, glycerol 20%, NP-40 1%, MgCl_2_ 1mM, EDTA 0.5mM, EGTA 0.1mM) for PBMC samples or 2) M-PER (Mammalian Protein Extraction Reagent) lysis buffer (ThermoFisher Scientific) for MM1.S, C7-14 and C1-15 cells, or 3) NP-40 lysis buffer (1% NP-40, 50mM Tris-HCl pH 8.0, 150mM NaCl) for co-immunoprecipitation. All lysis buffers were freshly supplemented with Halt protease and phosphatase inhibitor cocktail, EDTA-free (ThermoFisher scientific). Next, the protein concentration of the samples was measured via the Lowry method using the DC protein assay (Bio-Rad). 25μg (or less) of total protein was denatured, loaded on a SDS-PAGE gel, blotted on a nitrocellulose membrane (Bio-Rad), followed by standard antibody probing procedures (Santa Cruz Biotechnology).

As primary antibodies, we used: anti-GR (H300) (cat nr: sc-8992, Santa Cruz Biotechnology), anti-NCOA1 (SRC-1) (cat nr: 128E7, Cell Signaling). As loading control, we used the following primary antibodies: anti-GAPDH (cat nr: ab9485, Abcam), anti-GAPDH (cat nr: G8795, Sigma). As secondary antibodies, we used species-specific HRP-conjugated antibodies (cat nr: NA931, NA934, GE-Healthcare). To visualize results, we used Pierce ECL (Thermo Fisher Scientific), Westernbright Quantum or Sirus (Isogen) as chemiluminescent substrates and developed using X-Ray films (GE healthcare) or imaged on a ProXima 2850 imaging system (Isogen). WB results were quantified via band densitometric analyses using ImageJ. Relative protein levels were obtained by dividing the area under the curve (AUC) of the protein of interest by the AUC of the loading control.

### Peripheral blood mononuclear cell (PBMC) isolation

PBMCs were isolated from heparinized blood samples of healthy volunteers. Whole blood (20mL) was diluted with DPBS (ThermoFisher Scientific) in 1:1 ratio. As density centrifugation medium, Ficoll-Paque PLUS (GE healthcare) was used and 15mL was added to the SepMate tube (cat nr: 15460, 50mL format, StemCell Technologies) through the central hole of the SepMate insert. The diluted blood was added by pipetting it down the SepMate tube wall, followed by centrifuging the SepMate tube for 15’ at 1200g, room temperature (RT) with the brake on. The top layer containing the PBMCs and plasma is poured off in a fresh 50mL tube and is centrifuged for 8min at 300g, RT with the brake on. The supernatant is removed and the pellet is washed twice with RPMI1640 glutamax (Gibco, life technologies) supplemented with 2% fetal calf serum (Greiner bio-one), 100U/mL penicillin and 0.1mg/mL streptomycin (Gibco, life technologies) and centrifuged for 8min at 300g, RT with the brake on. Next, the cells were counted, transferred to a 6-well plate and induced for 2h with EtOH or Dex (1μM). Protein lysates were prepared using Totex lysis buffer.

### Microarray assay for real-time coregulator nuclear receptor interactions (MARCoNI)

MM1.S, C7-14, C1-15, A549 cells and PBMCs were induced for 2h with EtOH or Dex (1μM) and collected by washing twice with ice-cold phosphate buffered saline (PBS). Protein lysates were prepared by addition of M-PER lysis buffer (or Totex lysis buffer for PBMCs), and were placed in a thermomixer (Eppendorf) for 5’ at 1000rpm, RT. Next, the lysates were centrifuged together with the primary anti-GR (cat nr: sc-8992, Santa Cruz Biotechnology) and secondary anti-Alexa 488 antibody (cat nr: A-11070, ThermoFisher Scientific) for 1h at 20000g, 4°C. Next, 25μL of assay mix is prepared per sample and contains 10μL of lysate, 1μL DTT (0.05mM), 12.5μL 2x NR-buffer, 0.94μL anti-GR antibody (50nM for cell lines, 100nM for PBMCs) and was filled up to 25μL with Milli-Q water. The PamChip arrays (Pamgene) were placed in the Pamstation (Pamgene), operated by Evolve software, and blocked with 25μL StartingBlock buffer (ThermoFisher Scientific) per array. Then, 25μL assay mix/array is loaded, followed by washing steps, the addition of 25uL of anti-Alexa 488 (40nM for cell lines, 80nM for PBMCs) antibody, and final washing steps after which images of the arrays are taken at defined exposure times. The analysis of these images was done using BioNavigator software, which does automated spot-finding, quantifies the spots and determines the signal-over-background ratio. The resulting data was exported and further analyzed in R (see Statistical Analyses).

### Co-immunoprecipitation (co-IP)

Cells were induced as specified in the figure legends and lysed in NP-40 lysis buffer. Immobilized (using 2mg/mL BSA) Dynabeads (50μL bead slurry, cat nr: 11204D, ThermoFisher Scientific) were added to the lysate and rotated for 1h at 4°C. Using the Dyna-Mag 2 magnet (ThermoFisher Scientific) the supernatant (precleared lysate) was separated from the beads and the protein concentration was measured via the Lowry method (see above). Then, 150μg precleared lysate was combined with 5μL anti-GR antibody (cat nr: sc-8992, Santa Cruz Biotechnology) and rotated for 1h at 4°C. Immobilized Dynabeads (50μL bead slurry) were added to the mixture and rotated for another 2h at 4°C. The bead-mixtures were washed three times with NP-40 lysis buffer and were denatured for 5’ at 95°C using a thermomixer (Eppendorf). The samples were subjected to WB analysis and anti-NCOA1 (SRC-1) antibody (cat nr: 128E7, Cell Signaling) was used to assay the interaction between immunoprecipitated GR and NCOA1.

### Transfection

HEK293T were transfected with pSRC1a using jetPRIME (Polyplus), following the manufacturer’s instructions. Cells were collected 24h post-transfection, after which protein lysates (Totex) were prepared and WB was performed.

### Statistical analyses

Data analysis of the MARCoNI results was performed in R. The cutoff of the signal intensity was set at 10, and thus coregulators with a mean signal intensity lower than 10 were removed. Normalization of the data was performed using a local regression fit (loess), which subtracts a fitted value for each point from the observed value [59]. To this end, each technical replicate is normalized to a virtual reference (geometric mean of all replicates) and the degree of smoothing is controlled by a span parameter that is optimized to minimize the sum of deviations from the geometric mean. For the statistical analysis, an independent filtering procedure was applied to maximize hypothesis rejections and thus significant results [60]. It consisted of computing the variance for each coregulator across compared conditions, ranking the coregulators on variance from low to high and selecting the variance cutoff. In the next step, a Welch t-test, that does not assume equal variances, is used to compare the mean of two conditions. To correct for multiple testing, the false discovery rate (FDR) was used. Results were designated significant when the p-value (p) < 0.05.

Results are presented as scatter plots, in which the mean +/- standard error of the mean (SEM) are depicted. No statistical test was used to predetermine sample size, but we performed 3 (or more) biological replicates per MARCoNI experiment.

## SUPPLEMENTARY MATERIALS FIGURES AND TABLE



## Abbreviations

ALL: acute lymphoblastic leukemia
AML: acute myeloid leukemia
AP-1: activator protein-1
BC: breast cancer
CARM: co-activator associated methyltransferase 1
CBP: cyclic AMP response-element binding protein
CLL: chronic lymphoblastic leukemia
CREB: CBP-binding protein
DAX-1: dosage sensitive sex-reversal (DSS), adrenal hypoplasia congenita (AHC) critical region on the X-chromosome gene 1
EGF: epidermal growth factor
ER: estrogen receptor
GC: glucocorticoid
GR: glucocorticoid receptor
HAT: histone methyltransferase
Her2: human epidermal growth factor receptor 2
HMT: histone methyltransferase
JHD2C: probable JmjC domain-containing histone demethylation protein 2C
LCOR: ligand-dependent corepressor
MAPK: mitogen-activated protein kinase
MARCoNI: microarray assay for real-time coregulator nuclear receptor interactions
MM: multiple myeloma
PBMC: peripheral blood mononuclear cells
MOZ: monocytic leukemia zinc finger protein
NCOR: nuclear receptor corepressor
NFKBIB: nuclear factor-kappa-B inhibitor beta
nGRE: negative glucocorticoid response element
NR0B2: nuclear receptor subfamily 0 group B member 2
PGC-1ɑ: Peroxisome proliferator-activated receptor gamma coactivator-1ɑ
PIAS: protein inhibitor of activated STAT
PNRC2: proline-rich nuclear receptor coregulatory protein 2
PPAR: peroxisome proliferator activated receptor
PRAME: preferentially expressed antigen in melanoma
PTM: post-translational modification
NR: nuclear receptor
RIP140: receptor-interacting protein 140
SHP: small heterodimer protein
SMI: small molecule inhibitor
SMS: small molecule stimulator
SRC: steroid receptor coactivator
TF: transcription factor
TRIP3: thyroid receptor interacting protein 3
