# Supplementary material for: Coregulator profiling of the glucocorticoid receptor in lymphoid malignancies
Source: Oncotarget. 2017 Nov 30;8(65):109675–91. doi: 10.18632/oncotarget.22764 (PMC5752551; doi:10.18632/oncotarget.22764)
Supplement: Supplementary file 1 [file oncotarget-08-109675-s001.pdf]

Coregulator profiling of the glucocorticoid receptor in lymphoid malignancies

SUPPLEMENTARY MATERIALS

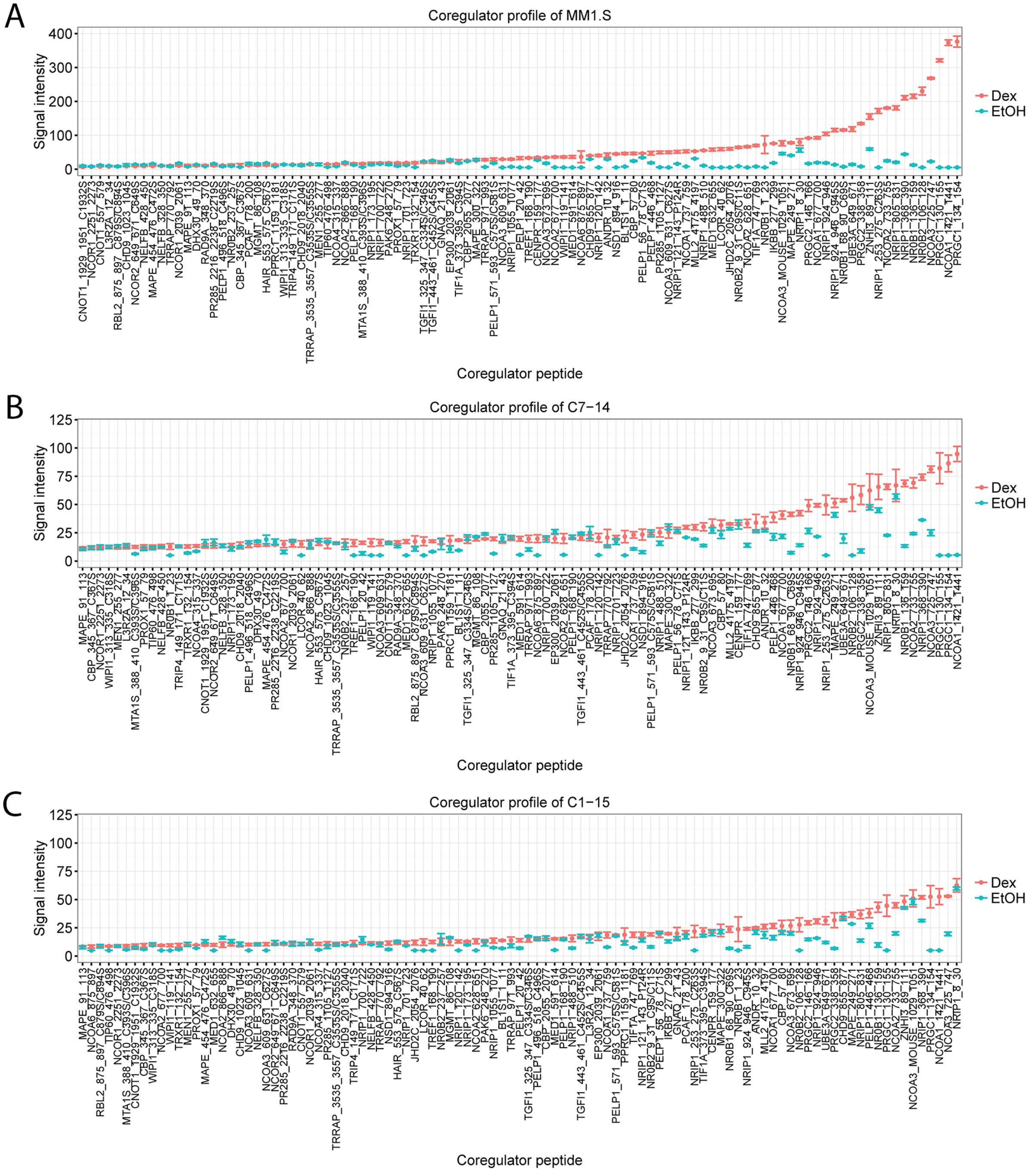

Supplementary Figure 1: Endogenous GR coregulator profile in MM and ALL cells. **(A)** MM1.S, **(B)** C7-14 and **(C)** C1-15 cells were treated for 2h with solvent or Dex (1µM). Protein lysates were prepared and subjected to MARCoNI analyses. The coregulator plots represent the mean signal intensity +/- the standard error of the mean (SEM) of 3 biological replicates. Coregulators were ranked according to Dex response.

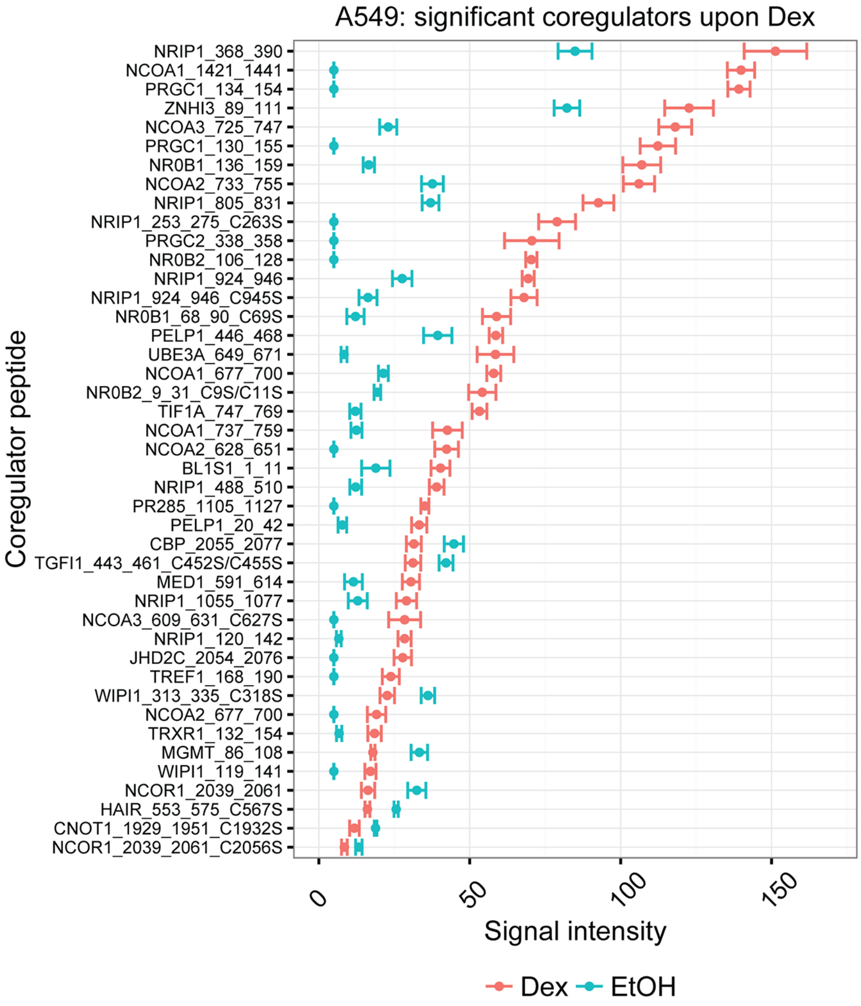

Supplementary Figure 2: Significant coregulators upon Dex treatment in A549 cells. (A) A549 cells were treated for 2h with solvent or Dex (1µM). Protein lysates were prepared and subjected to MARCoNI analyses. The coregulator plot represents the mean signal intensity +/- SEM of 4 biological replicates. Coregulators responding statistically significant to Dex treatment are displayed and were ranked according to Dex response. Statistical analysis was performed in R, using Welch t-tests corrected for multiple testing using the false discovery rate (FDR, 5%).

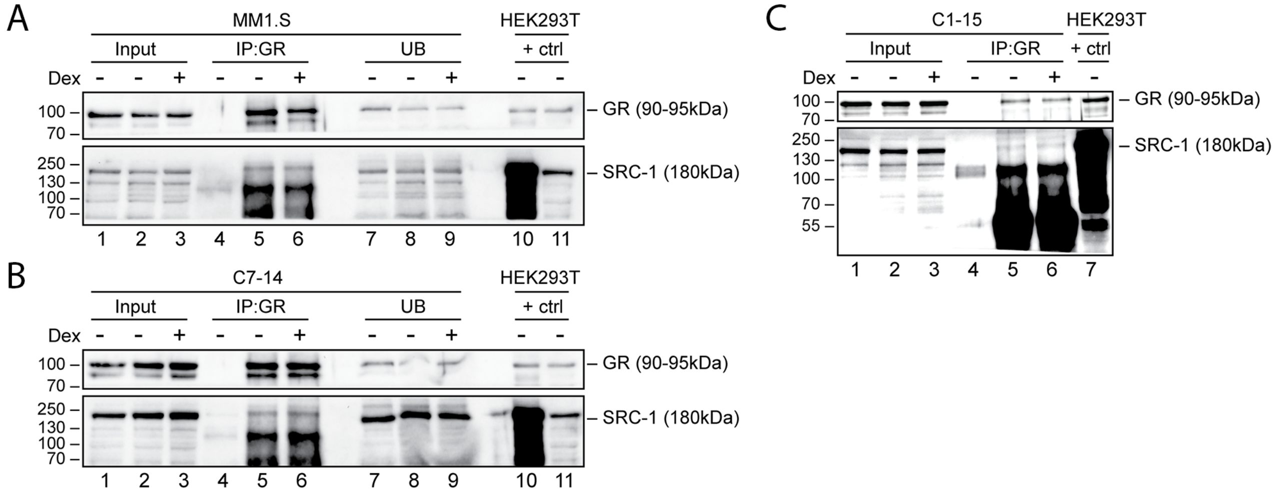

Supplementary Figure 3: Endogenous GR co-immunoprecipitation in MM and ALL cells. (A) MM1.S, **(B)** C7-14, **(C)** C1-15 cells were treated for 2h with solvent or Dex (1µM). Protein lysates were prepared and endogenous GR co-immunoprecipitation was performed, followed by WB analysis to detect the protein levels of GR (90-95kDa) and SRC-1 (180kDa). As a negative control for IP, a non-specific antibody was used (lane 4, 7). Unbound fractions (UB) are presented (A, B: lane 7, 8 and 9). As a positive control for the SRC-1 antibody, HEK293T lysate (A, B: lane 11) or HEK293T cells overexpressing SRC-1 (A, B: lane 10, C: lane 7) were used. WB results are representative for 2 independent experiments.

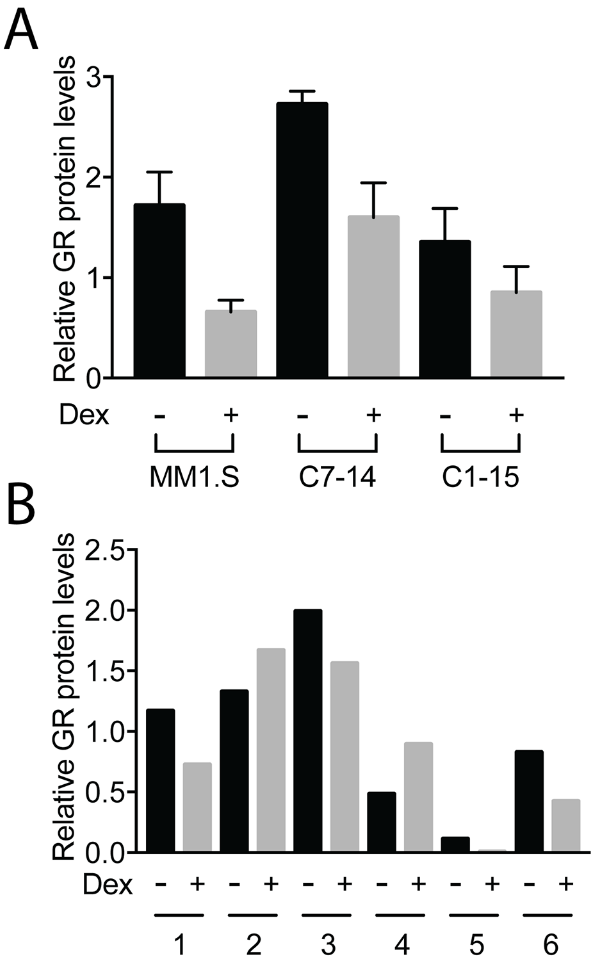

Supplementary Figure 4: Quantification WB results. WB densitometric analysis was performed using ImageJ. The GR protein levels were normalized to GAPDH loading control levels. **(A)** Relative GR protein levels in MM1.S, C7-14 and C1-15 cells. **(B)** Relative GR protein levels in PBMCs.

Supplementary Table 1: Significant coregulators upon Dex treatment for A549 cells. A Welch t-test was used identify coregulators that significantly respond to Dex treatment. The p-values were corrected for multiple testing using FDR (5%).

A549

coregulator	p-value
PRGC1_134_154	0.00103
NCOA3_725_747	0.00103
NR0B2_106_128	0.00103
TIF1A_747_769	0.00103
NCOA1_677_700	0.00103
NCOA1_1421_1441	0.00119
NCOA2_733_755	0.00119
NRIP1_924_946_C945S	0.00131
NRIP1_924_946	0.00131
HAIR_553_575_C567S	0.00131
PR285_1105_1127	0.00155
NRIP1_488_510	0.00155
PRGC1_130_155	0.00241
NR0B1_136_159	0.00241
NRIP1_805_831	0.00241
NR0B1_68_90_C69S	0.00241
PELP1_20_42	0.00257
NRIP1_120_142	0.00507
NRIP1_253_275_C263S	0.00643
NCOA2_628_651	0.01232
NRIP1_368_390	0.01455
UBE3A_649_671	0.01455
NR0B2_9_31_C9S/C11S	0.01486
MED1_591_614	0.01510
JHD2C_2054_2076	0.01698
PRGC2_338_358	0.02014
NCOA1_737_759	0.02014
NCOR1_2039_2061	0.02176
WIPI1_313_335_C318S	0.02222
TREF1_168_190	0.02308
WIPI1_119_141	0.02353
ZNHI3_89_111	0.02562
MGMT_86_108	0.02562
TRXR1_132_154	0.02562
BL1S1_1_11	0.03346
NRIP1_1055_1077	0.03625
NCOR1_2039_2061_C2056S	0.04532
PELP1_446_468	0.04607
CBP_2055_2077	0.04607
NCOA2_677_700	0.04607
TGFI1_443_461_C452S/C455S	0.04607
CNOT1_1929_1951_C1932S	0.04607
NCOA3_609_631_C627S	0.04999
